# The second-tier status of fragile X syndrome testing for unexplained intellectual disability/global developmental delay in the era of next-generation sequencing

**DOI:** 10.3389/fped.2022.911805

**Published:** 2022-07-22

**Authors:** Wen Zhang, Dong Li, Nan Pang, Li Jiang, Baomin Li, Fanghua Ye, Fang He, Shimeng Chen, Fangyun Liu, Jing Peng, Jinghua Yin, Fei Yin

**Affiliations:** ^1^Department of Pediatrics, Xiangya Hospital, Central South University, Changsha, China; ^2^Hunan Intellectual and Developmental Disabilities Research Center, Pediatrics, Changsha, China; ^3^Clinical Research Center for Children Neurodevelopmental Disabilities of Hunan Province, Xiangya Hospital, Central South University, Changsha, China; ^4^Department of Neurology, Children's Hospital Affiliated to Chongqing Medical University, Chongqing, China; ^5^Department of Pediatrics, Qilu Hospital of Shandong University, Jinan, China; ^6^Department of Pathophysiology, Xiangya Hospital, Central South University, Changsha, China

**Keywords:** fragile X syndrome, intellectual disability, global developmental delay, neurodevelopmental, genetic testing

## Abstract

**Objective:**

Although many unexplained intellectual disability/global developmental delay (ID/GDD) individuals have benefited from the excellent detection yield of copy number variations and next-generation sequencing testing, many individuals still who suffer from ID/GDD of unexplained etiology. In this study, we investigated the applicability of fragile X syndrome (FXS) testing in unexplained ID/GDD individuals with negative or absent genetic testing.

**Methods:**

In this study, we used the triplet repeat primed polymerase chain reaction to evaluate the value and application of fragile X testing in unexplained ID/GDD individuals with negative or absent genetic testing (*n* = 681) from three hospitals.

**Results:**

Of the 681 ID/GDD individuals with negative or absent genetic testing results detected by FXS testing, 12 men and one woman were positive. This corresponded to a diagnostic yield of 1.9% for FXS testing in our cohort. All FXS individuals had either a family history of ID/GDD or suggestive clinical features. The detection yield of FXS testing in ID/GDD individuals who completed genetic testing (2.70%, 12/438) was significantly higher than in individuals without any genetic testing (0.40%, 1/243).

**Conclusions:**

This is the first report of FXS testing in ID/GDD individuals who lacked previous genetic testing, which promotes standardization of the FXS diagnostic process. These results highlight the utility of FXS testing of unexplained ID/GDD individuals with negative results from standard genetic testing. In the era of next-generation sequencing, FXS testing is more suitable as a second-tier choice and provides clinicians and geneticists with auxiliary references for tracing the etiology of ID/GDD.

## Introduction

Fragile X syndrome (FXS), an X-linked neurodevelopmental disorder (NDD) with incomplete penetrance, is the most common monogenic cause of intellectual disability (ID) and autism (1). In most cases, unstable amplification and aberrant methylation of CGG repeats near the promoter in the fragile X mental retardation 1 (*FMR1*) results in a reduction or deletion of fragile X mental retardation protein (FMRP) (2). FMRP plays a primary role in human cortical development and is a key regulator of genes associated with ID and autism spectrum disorders (3, 4). Decreased FMRP has been shown to adversely affect neurodevelopment and leads to many cognitive impairment phenotypes.

Fragile X syndrome has been studied for decades, but relatively little basic medical and clinical research has been conducted on FXS in China. Worldwide, the prevalence of FXS has been shown to be ~1 in 4,000–7,000 in male and 1 in 8,000–11,000 in female (5–7). Countries with larger Asian populations, such as Taiwan and Japan, have a significantly lower prevalence of FXS than other Western countries. Several previous studies have reported the prevalence of FXS in Chinese populations (8–11), with the detection yield of FXS in ID and global developmental delay (GDD) having been reported to range from 0.6 to 2.8%, which generally is lower than in other Western countries. To date, no large-scale epidemiological studies on FXS have been conducted in China. It is thus imperative to increase knowledge on FXS in Chinese populations and to standardize the diagnostic process.

Although large-scale screening programs have been useful for determining the prevalence of FXS in Chinese populations, the cost-effectiveness of FXS screening has been questioned. In this era of next-generation sequencing (NGS), clinical genetic testing methods have been revolutionized in clinical settings. Copy number variations (CNV) and NGS testing also have emerged as excellent options for identifying genetic causes of NDDs (12–14), which expands our understanding of the pathogenesis of NDDs. It is a cruel reality for some individuals with ID/GDD, however, that clinicians still cannot find the etiology after genetic testing (15). The inability to detect repeats and methylation of CGG repeats in the *FMR1* gene is a significant deficiency of NGS and CNV testing. The value of FXS testing in this genetically negative population, however, remains unclear.

The purpose of this study was to investigate the value of FXS testing in individuals with negative genetic results. In this study, we performed the triplet repeat primed polymerase chain reaction (TP-PCR) for FXS testing in 681 unknown ID/GDD individuals with negative or absent genetic results. Although this method has been applied in other diseases previously (16, 17), this is the first report of FXS testing in individuals with negative genetic results. These results highlight the utility of FXS testing in individuals with unexplained ID/GDD and a history of negative genetic testing, especially in individuals with positive clinical features or family history. FXS testing thus can be recommended as second-tier testing strategy for clinicians and ID/GDD individuals for tracing the genetic etiology of neurodevelopmental delays, which promotes standardization of the FXS diagnostic process.

## Materials and methods

### Individuals with ID/GDD

The subjects of this study were children with unexplained ID/GDD who visited Xiangya Hospital at the Central South University, Qilu Hospital of Shandong University, and Children's Hospital of Chongqing Medical University from January 2017 to December 2019. The severity of ID/GDD was scored using the Wechsler Intelligence Scale for Children or the Gesell developmental scale. Based on developmental quotient scores, the severity of ID/GDD was classified as mild (55–70), moderate (40–54), severe (25–39), and profound (<25). These individuals underwent neuroimaging (magnetic resonance imaging or computed tomography), screening for metabolic disorders (urine organic acid analysis using gas chromatography-mass spectrometry, acylcarnitine analysis, and the detection of amino acid levels in the blood by tandem mass spectrometry), and chromosomal karyotype to exclude acquired injuries, metabolic disorders, trisomy, and other identifiable causes of ID.

**Figure 1:**
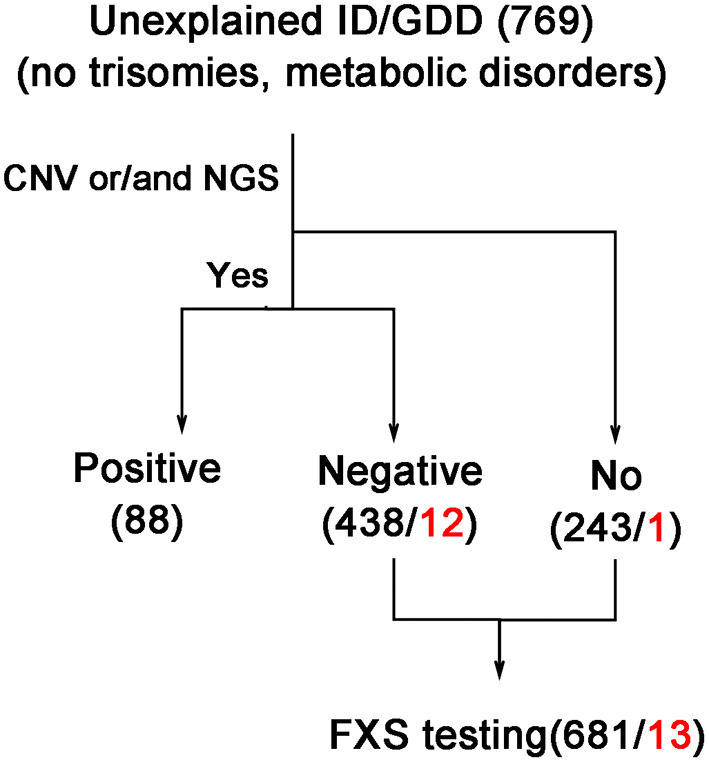
Strategy of selection for individuals with ID/GDD who were tested by FXS testing. The black and red numbers indicate the total and positive numbers, respectively. CNV, copy number variations including chromosome microarray analysis and CNV-seq detection; ID/GDD, intellectual disability/global developmental delay; NGS, next-generation sequencing; including gene panel, clinical exome sequencing, and whole exome sequencing examination.

We initially included 769 cases of unexplained ID/GDD. Except for individuals whose clinical presentation was highly suggestive of a specific genetic diagnosis that would be recommended for targeted genetic testing, other individuals with negative neuroimaging, metabolic screening, and negative karyotypes were first recommended for CNV or NGS testing. However, the degree of completion of genetic testing varied widely, which was in line with the different preferences of families in clinical practices. Therefore, in this retrospective study, we excluded 88 individuals with ID/GDD of known etiology confirmed by genetic testing. Of these, 681 individuals (550/84/47 from each hospital) were assessed using FXS testing (details in Table 1). Figure 1 illustrates the selection strategy. This study was approved by the Institutional Research Ethics Committee of Xiangya Hospital, Central South University. The guardians of each individual provided informed consent for genetic testing.

**Table 1:** General information of unexplained etiology ID/GDD individuals (*n* = 681).

**Clinical characteristics**	**ID patients** ***n*** **(%)**	**FXS positive** ***n*** **(%)**
**Sex**
Male	505 (74.26)	12 (92.3)
Female	176 (25.64)	1 (7.69)
**Age**
<1 year	55 (8.08)	0 (0)
≥1, ≤ 3 years	164 (24.08)	3 (23.08)
>3, ≤ 6 years	244 (35.83)	3 (23.1)
>6, ≤ 18 years	218 (32.01)	7 (53.8)
**ID/GDD severity**
Mild	203 (29.81)	3 (23.1)
Moderate	214 (31.42)	3 (23.1)
Severe	203 (29.1)	4 (30.8)
Profound	61 (8.96)	2 (15.9)
**Isolated IDD**	342 (50.22)	7 (53.8)
**Non-isolated IDD**	339 (49.78)	6 (46.6)
Epilepsy	257 (37.74)	3 (23.8)
Autistic features or autism	56 (8.22)	0 (0)
ADHD	26 (3.82)	3 (23.1)
**Auxiliary examinations**
Neuroimaging	440 (64.61)	
GC/MS analysis	443 (65.05)	
Karyotype analysis	383 (56.24)	
^#^CNV testing	397 (58.30)	
^*^Next-generation sequencing	200 (29.37)	

# *Including chromosome microarray analysis and CNV-seq detection*.

* *Including gene panel, clinical whole-exome sequencing and whole-exome sequencing. ADHD, Attention deficit and hyperactivity disorder; CNV, Copy number variations; ID/GDD, Intellectual disability/Global developmental delay; GC/MS, Gas chromatography-mass spectrometry*.

### FXS testing

We used 3–4 ml of peripheral blood collected in an ethylene diamine tetraacetic acid anticoagulant tube for genomic DNA extraction using the standard phenol-chloroform method. The DNA concentration was measured using a NanoDrop™ spectrophotometer. (Thermo Scientific, USA) The number of CGG repeats in *FMR1* was measured by TP-PCR using the Amplide X FMR1 PCR Kit (Asuragen, USA) following the manufacturer's protocol. PCR was performed using an ABI GeneAmp PCR 9700 thermal cycler (Applied Biosystems, USA). Amplicons were sized on an ABI 3500xl Genetic Analyzer (Applied Biosystems) and analyzed using GeneMapper 4.0 software (Applied Biosystems). Based on the CGG repeat length, FXS testing results were classified as full mutation (≥200 repeats), pre-mutation (55–200 repeats), intermediate or gray zone (45–54 repeats), and normal ( ≤ 45 repeats). Full mutation mosaic referred to an individual with subpopulations of full mutation, and other repeat with permutations, near-normal, or normal CGG repeat lengths.

### Statistical analyse

Groups were compared using the chi-square test, Fisher's exact test was used where 20% or more of the gird in the chi-square table were expected to count <5. Statistical analysis was performed using SPSS (version 18.0, USA) software. A value of *P* < 0.05 was considered statistically significant. Bonferroni correction was used for multiple tests.

## Results

### Results of FXS testing in individuals with unexplained etiology

To clarify the potential etiology of unexplained IG/GDD and the utility of FXS testing as a complementary testing after negative genetic testing, we assessed 681 unexplained ID/GDD individuals with negative or absent genetic testing for FXS by TP-PCR (details in Table 1). Individuals with chromosomal abnormalities (trisomy), metabolic disorders, and positive genetic testing were excluded from this study. Among them, 505 individuals (74.15%) were males and 176 (25.84%) were females. The age of the individuals and the severity of ID/GDD assessment are detailed in Table 1. The ratio of isolated ID to non-isolated ID was 1.01, and epilepsy and attention deficit and hyperactivity disorder (ADHD) were common phenotypes. Among these 681 individuals, only 12 male and one female were found to have more than 200 CGG repeats in FXS testing (Table 2). In addition, we detected two pre-mutations (two males) and five intermediate mutations (three males and two females). This corresponded to a diagnostic yield of 1.9% for FXS testing in this cohort.

**Table 2:** Results of fragile X testing among unexplained etiology ID/GDD individuals.

	**Male**	**Female**	**Total**
**Positive (*n* = 13)**
Full mutation	11	0	11
Full mutation mosaic	1	1	2
**Negative (*n* = 668)**
Pre-mutation	2	0	2
Intermediate	3	0	3
Normal	488	175	663

Of these 13 FXS individuals, 11 were full mutation and two were full mutation size mosaics (Table 2). All were diagnosed with ID at the age of 1 year or older. In addition, the severity of neurodevelopmental impairment varied among the 13 FXS individuals. Three cases were mild, three cases were moderate, four cases were severe, and two cases were profound ID. There were seven cases of isolated ID, three cases with epilepsy, and three cases with ADHD. Retrospective analysis showed that all 13 FXS individuals had either family history or suggestive clinical features. The yield of FXS testing was significantly higher in individuals with family history only (14.3%) and both family history/clinical features (21.4%) than in individuals without clinical features/family history (0%) (Table 3). Notably, six of the 10 with a positive family history were suspected of X-linked ID. Moreover, four of the individuals were two pairs of siblings. In both families, the mothers of the proband were pre-mutation carriers, and no primary ovarian insufficiency was recorded until a recent follow-up was conducted. These results suggested that family history or clinical features provide a crucial basis for recommending FXS testing.

**Table 3:** Comparison of the yield of fragile X testing in unexplained ID/GDD individuals with or without clinical features and family history (*n* = 681).

	**FXS negative**	**FXS positive**	***P-*** **value** ^*^
Family history only *n* (%)	24 (85.7)	4 (14.3)	Fisher *P* = 0.000
Clinical feature only *n* (%)	169 (98.8)	3 (1.7)	Fisher *P* = 0.021
Both family history and clinical features *n* (%)	22 (78.6)	6 (21.4)	Fisher *P* = 0.000
Neither clinical features nor family history *n* (%)	453 (100)	0 (0)	

* *Compared with “Neither clinical features nor family history” group, respectively. P < 0.0083 was considered to be significant, based on the Bonferroni correction*.

### Comparison of the yield of FXS testing in unexplained etiology ID/GDD individuals with negtive or absent genetic evidence

**Figure 2:**
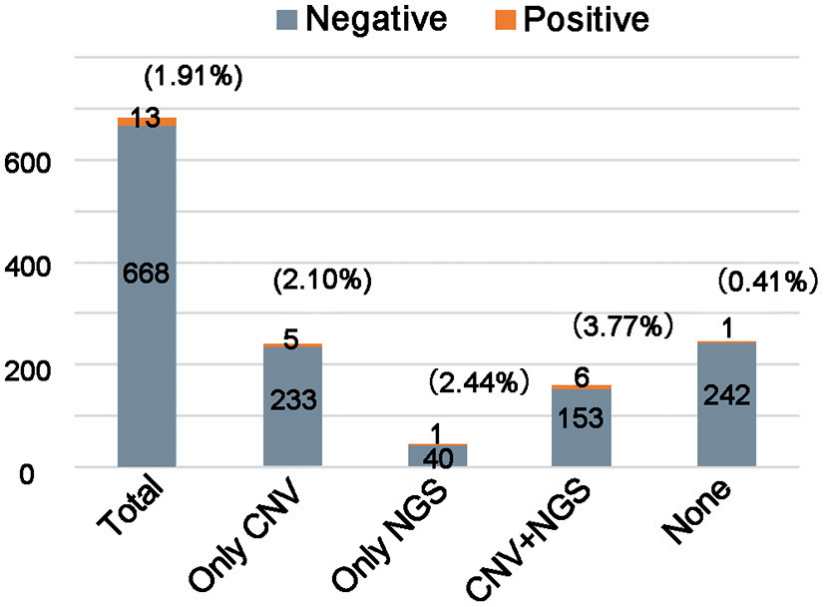
Comparison of the yield of fragile X testing in unknown etiology ID/GDD patients with or without genetic examination. The number of individuals analyzed is shown in each column.

Affected by objective factors such as level of education and economics, the situation of families in choosing detection methods is often complex. Many parents will selectively complete the CNV or/and NGS testing. We counted the detection yield of FXS testing in the different genetic testing backgrounds (Figure 2). Among these 681 individuals, 238 individuals had completed CNV testing only, 41 individuals had completed NGS testing only, and 159 individuals had completed both CNV and NGS testing The detection yield of FXS testing was 3.77% (6/159) in those who were negative for both CNV and NGS testing. If we limited FXS testing to the CNV and NGS testing negative population, and further excluded individuals with neither a positive family history nor clinical features (98 individuals), the theoretical yield of FXS testing could be increased to 9.84% (6/61). Such an estimate of detection yield tended to overlook the proportion of FXS individuals without clinical features or positive family history. The detection yield of FXS testing in those who completed either CNV or/and NGS testing (2.70%, 12/438) was significantly higher than in the population without any genetic testing (0.40%, 1/243). These results suggested that FXS testing is a meaningful second-tier tool for diagnosing unexplained ID/GDD.

## Discussion

Copy number variations and FXS testing have been used as first-tier options to identify genetic causes for many children with unknown etiology ID/GDD over the past decade (12, 18). The introduction of NGS technology in recent years, however, has revolutionized the field of genetic diagnosis. In 2021, the American College of Medical Genetics and Genomics (ACMG) strongly recommended that exome and genome sequencing be considered as a first- or second-tier tool for diagnosis of ID/GDD (19). Although many individuals are benefiting from the excellent detection yield of CNV and NGS testing (20, 21), many other individuals still suffer from ID/GDD of unknown etiology. Thus, in this, we investigated the applicability of FXS testing in unexplained ID/GDD individuals with negative or absent genetic testing. This was the first report of FXS testing in individuals with negative genetic test results. Our findings support the recommendation of FXS testing as second-tier tool for unexplained ID/GDD individuals.

The yield of FXS testing was 1.9% (13/681) in our cohort. In fact, we excluded 88 CNV- or NGS-positive individuals during the screening progress, which led to an overestimation of FXS testing yield for unexplained ID/GDD. Later in the study, we also performed TP-PCR on 88 genetic testing-positive individuals, and all CGG repeats assessed were normal. This result implied that the theoretical yield of FXS testing maybe 1.7% (13/769) to 1.9% (13/681) in general ID/GDD individuals who remain etiologically unidentified after neuroimaging, metabolic screening, and karyotyping detection. In our research, the yield of FXS testing is 0.57% (1/176) in females, which is similar to the reported studies in western counties (22, 23). These results confirmed the gender, ethnic, and region differences in the prevalence of FXS prevalence. The other reasons for fluctuations in the yield of FXS testing in individuals with ID/GDD may include differences in race, environment, education, or medical care level (24). As in our ID/GDD individual selection strategy, the independent definitions of individuals with unexplained ID/GDD in each study also may contribute to differences in our detection yield. For example, in Chen's study (8), the yield of full mutations was only 0.9% (5/540) after excluding mild ID and ID-related genomic copy number variants or genomic mutations in this population. In Borch's study of a population of 2,486 neurodevelopmental disorders, the yield of FXS testing fluctuated between 0 and 2.5% in different segments, which is the largest population investigated in similar circumstances to date (22). Overall, the detection yield of FXS testing was still much lower than that of CNV (10–25%) and NGS (20–30%) testing in individuals with ID (20, 21). More clinicians and genetic counselors are thus recommended to prioritize NGS and CNV testing in the absence of strong evidence of FXS testing.

The coverage rates of CNV and NGS testing in our cohort were ~62.01% (477/769) and 35.11% (270/769). Except for individuals whose clinical presentation was highly suggestive of a specific genetic diagnosis that would be recommended for targeted genetic testing, other individuals with negative neuroimaging, metabolic screening, or negative karyotypes were first recommended for CNV and/or NGS testing. The ultimate practical choice for CNV and NGS testing, however, depends on many factors, including health-care system policies, insurance status, individual parental preferences, and economic levels. Another harsh reality is that a large proportion of individuals with ID/GDD remain unidentified after NGS and/or CNV testing. Our study found a higher yield of FXS testing in individuals who completed at least one of the CNV or NGS test (2.70%, 12/438) than in individuals without genetic testing (0.40%, 1/243). When we further narrowed the testing to individuals with either positive clinical features or family history, and both negative CNV/NGS testing, the theoretical yield of FXS testing was 9.84% (6/61), which was much higher than the detection rate in general ID/GDD individuals (22). This result suggested that it was meaningful to use FXS testing as a second-tier test for unexplained ID/GDD. Although this can result in a delay of 1–2 months in diagnosis. To date, although some experimental and clinical studies have made gratifying advances, effective treatments for FXS remain limited (25). This diagnostic delay does not fundamentally affect the care of FXS, except in extraordinary conditions, such as preparation/termination of pregnancy, the intervention of assisted reproductive technology, or pre-implantation examination. Therefore, we recommend FXS testing in ID/GDD individuals with negative NGS or CNV testing.

Considering that if FXS testing is transitioned to be the second-tier recommended testing of ID/GDD, the diagnosis of FXS with mild phenotypes will not be omitted. Because some clinical centers are not familiar with FXS, the potentially positive clinical signs are often inadvertently ignored. The age of diagnosis is a crucial consideration in determining these clinical features. For example, the large testicles, which are more prevalent among the clinical features of FXS, have not been described uniformly in previous reports. Merryash's study reported that 13 of 15 individuals ages 18–69 years old had large testicles (26). In contrast, none of the 14 pre-pubertal males in the study had large testicles (27). Suggestive FXS facial features, such as a long face, large ears, and prominent jaw, are also very common in CNV variants. In addition, considering that most individuals with FXS require lifelong care management and treatment, transitioning FXS testing to be a second-tier test could effectively utilize limited laboratory human resources and promote a rational allocation of health-care system resources so that more individuals and families can benefit from them. Moreover, as FXS testing is relatively inexpensive, even with a lower yield compared with CNV and NGS testing, it still plays a role in appropriate individuals, such as female carriers at risk for developing primary ovarian insufficiency.

In terms of diagnostic technology, TP-PCR and Southern blotting methods are still the gold standard for the identification of amplified *FMR1* alleles and quantification of CGG repeats (24). NGS can identify point mutations and insertional deletion variants that account for ~1% of FXS (28). The influx of new technologies such as short-read NGS technologies (29) and single-molecule real-time long-read sequencing (30), can compensate for some of the deficiencies of TP-PCR and Southern blot assays. With the reduction of error rate and cost, more bioinformatics tools will be used for clinical applications, which can effectively improve the diagnosis yield of FXS in the future.

The detection yield of FXS testing in our study was not representative of the prevalence of FXS in generalized individuals with ID/GDD, and the number of subjects in studies like this still needs to be expanded. Factors such as region, race, gender, and study sample size may affect the detection yield of FXS testing. The description of physical signs and deformity morphology of ID/GDD individuals relies too much on the judgment of clinicians. Improving an FXS pre-detection checklist may effectively reduce the bias caused by these objective factors. In addition, the complexity of an individual's preference for clinical detection causes a lot of trouble when categorizing data. A prospective cohort study with a larger number of samples will hopefully address these issues.

In 2021, ACMG published their guidelines for exome and whole-genome sequencing of childhood ID and laboratory technical standards for FXS (19, 24), and the priority of FXS testing in children with unexplained ID/GDD was downgraded. In this study, we used TP-PCR to evaluate the value and application of FXS testing in individuals with unexplained ID/GDD with negative or absent genetic testing. These results highlighted the utility of FXS testing in individuals with negative genetic test results, especially in individuals with positive clinical features or family histories. In the era of NGS, FXS is more suitable as a second-tier diagnostic choice, providing pediatricians and geneticists with auxiliary references for tracing the etiology of ID/GDD.

## Data availability statement

The original contributions presented in the study are included in the article/supplementary material, further inquiries can be directed to the corresponding author/s.

## Ethics statement

The studies involving human participants were reviewed and approved by Institutional Research Ethics Committee of Xiangya Hospital, Central South University. Written informed consent to participate in this study was provided by the participants' legal guardian/next of kin.

## Author contributions

FYi and WZ designed research. WZ, NP, FH, JP, JY, and FYi involved in data analysis. WZ, NP, JY, and FYi wrote the manuscript. WZ, DL, NP, LJ, BL, FYe, FH, SC, FL, JP, JY, and FYi performed research, read, edited, and approved the manuscript. All authors contributed to the article and approved the submitted version.

## Funding

This work was supported by the National Natural Science Foundation of China (81771408 and 81701541), the Hunan Key Research and Development Program (No. 2019SK2081), and the Natural Science Foundation of Hunan Province, China (No. 2021JJ40986).

## Conflict of interest

The authors declare that the research was conducted in the absence of any commercial or financial relationships that could be construed as a potential conflict of interest.

## Publisher's note

All claims expressed in this article are solely those of the authors and do not necessarily represent those of their affiliated organizations, or those of the publisher, the editors and the reviewers. Any product that may be evaluated in this article, or claim that may be made by its manufacturer, is not guaranteed or endorsed by the publisher.
